# Vitamin D serum levels and vitamin D receptor *FokI*polymorphism on tuberculosis children in Palembang, Indonesia

**DOI:** 10.1186/1687-9856-2013-S1-P202

**Published:** 2013-10-03

**Authors:** Ariesti Karmila, Aditiawati, Muhammad Nazir, Kiagus Yangtjik, Yuwono

**Affiliations:** 1 Department of Child Health, Medical School, University of Sriwijaya/Mohammad Hoesin Hospital Palembang Indonesia; 2 Department of Microbiology, Medical School, University of Sriwijaya/Mohammad Hoesin Hospital Palembang Indonesia

## Aim

The role of vitamin D on host immunity defense against tuberculosis infection has long been known. Deficiency vitamin D and vitamin D receptor polymorphism are strongly associated with the susceptibility of tuberculosis in four seasons countries. As a country with sufficient sunlight, the incidence of tuberculosis in Indonesia remains high. The aim of this study is to asses the association of vitamin D serum level and vitamin d receptor *FokI*polymorphism with the incidence of tuberculosis in children at Palembang, Indonesia.

## Method

A case control study was conducted at Child Health Department Mohammad Hoesin Hospital Palembang during November 2011 - April 2012. Case group consisted of children suffering tuberculosis disease while control group are healthy children who had been sensitized to tuberculosis provenwith a positive tuberculin test. Vitamin D (1,25(OH)_2_D_3_/calcitriol) serum level was measuredby using IDS 1,25-Dihydroxy Vitamin D EIAkit and VDR *FokI*polymorphism was identified through RFLP analysis. A bivariate and multivariate analysis were performed with *p* < 0,05 and CI 95%.

## Result

Sixty subjects were divided equally to case and control groups. The mean of calcitriolserum level in case group was lower compared to control eventhough still in normal level range (105,50 ± 66,86 pmol/Lvs162,90±52,86 pmol/L*p*=0,001). We found nine subjects with calcitriol deficiency, 8 (26,7%) in children with tuberculosis disease and 1 (3,3%) in children without tuberculosis disease (OR 10,54; 95% CI 1,22-90,66). The incidence of VDR *FokI*polymorphism is 93,4% in case group and 73,3% in control group (OR 5,0; 95% CI 0,9-26,4). No significant association was found between calcitriolserum level and VDR*FokI*polymorphism (*p*=0,999).

## Conclusion

Vitamin D (calcitriol) deficiency and low serum level are associated with higher risk of tuberculosis in children at Palembang, Indonesia. Polymorphism *FokI*in VDR gene also contribute to the susceptibility of tuberculosis. Our data supports that vitamin D also has a contribution in susceptibility to tuberculosis infection even in a country with sufficient sunlight exposure.

